# COVID-19: Review of Epidemiology and Potential Treatments Against 2019 Novel Coronavirus

**DOI:** 10.15190/d.2020.5

**Published:** 2020-04-26

**Authors:** Hasnain Jan, Shah Faisal, Ayyaz Khan, Shahzar Khan, Hazrat Usman, Rabia Liaqat, Sajjad Ali Shah

**Affiliations:** ^1^Department of Biotechnology, Quaid-i-Azam University, Islamabad, Pakistan; ^2^Department of Biotechnology, Bacha Khan University, Charsadda, Pakistan; ^3^Department of Microbiology, Abdul Wali Khan University, Mardan, Pakistan; ^4^Department of Botany, Bacha Khan University, Charsadda, Pakistan

**Keywords:** COVID-19, SARS-CoV-2, MERS, Morbidity, Chloroquine, Rendesivir.

## Abstract

An epidemic of extreme respiratory deterrence, pneumonia and shortness of breath, the SARS-CoV-2 viral infection began in Wuhan, Hubei Province, China in December 2019, and rapidly spread across China and beyond, with human to human transmission. On February 12, 2020, World Health Organization officially named the new coronavirus disease as coronavirus disease 19 (COVID-19). Most COVID-19 patients were diagnosed with pneumonia and many were treated using Chinese medicines and other secondary therapies. As of April 22, 2020, the total figure of infected patients has crossed 2.6 million people worldwide with over 180,000 deaths and 700,000 patients that have recovered. Preliminary reports suggest that certain drugs, such as chloroquine and antiviral nucleotide analogues such as remdesivir, which inhibit viral replication, can target the new coronavirus, although their usefulness in the clinic is still under debate. An expert US committee developed the US NIH guidelines for COVID-19 treatment, which was just released and will be regularly updated. This manuscript reviews the epidemiology, etiology, mortality, COVID-19 clinical symptoms, and potential therapeutic drugs, while highlighting the seriousness and damage-induced by SARS-CoV-2.

## SUMMARY

*1. Introduction*

*2. Etiology*

*3. Epidemiology and recent figures*

*4. Incubation period for COVID-19*

*5. COVID-19 mortality rate*

*6. COVID-19 symptoms*

*7. Treatment options for SARS-CoV-2 infection*

*8. Conclusion*

## 1. Introduction

In December 2019, Wuhan in the Hubei province of China announced an active epidemic of pneumonia associated with a novel coronavirus, identified as the severe acute respiratory syndrome coronavirus 2 (SARS-CoV-2)^1,2^. Over the next few weeks, infections spread throughout China and elsewhere in the world^3,4^. Chinese media, clinical and science institutions have reacted swiftly, such that the latest virus is identified and the viral genome sequence is shared quickly worldwide^2^. On January 30, 2020 the World Health Organization (WHO) announced an outbreak of Public Health Emergency of International Concern (PHEIC)^5^.

On February 12, 2020, WHO named the disease coronavirus disease 2019 (COVID-19) caused by the novel coronavirus^5^. A consortium of foreign experts collaborating with Chinese colleagues of various specializations has sought to hold on this outbreak^6^. The first infected cases were associated with a food market in Wuhan^1^. Coronaviruses have enveloped, positive sense single-stranded RNA genome, infecting humans and a wide range of animals. They were for the first time characterized and cultured by Tyrell and Bynoe in 1966, from patients with flu and common cold^7^. They were known as coronaviruses, based on their morphology as spherical virions with a central shell and surface projections identical to a solar corona (Latin: corona means crown)^8^. There are four separate subfamilies, the alpha, beta, gamma, and delta coronaviruses. Alpha and beta coronaviruses appear to come from mammals, especially from bats, whereas gamma and delta coronaviruses derive from pigs and birds^9^. The genome length ranges from 26kb to 32kb. Among the seven subtypes of coronavirus that may have the ability to infect humans the beta-coronavirus is considered the most dangerous; this is the one that causes significant morbidity and mortality in humans. SARS-CoV-2 virus belongs to the genus beta-coronavirus^9,10^.

**Figure 1:**
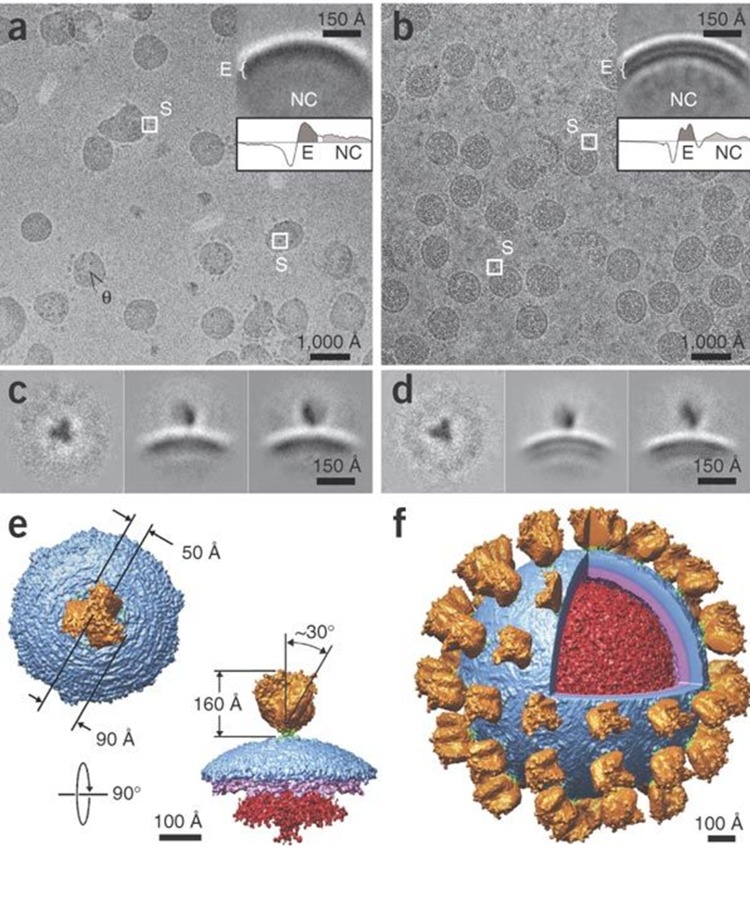
Figure 1: SARS-CoV structure (a, b) Microscopic images at 5 micron indicate NC for nucleocapsid, S for spikes and E for envelope (c, d) Two dimensional class averages data from a and b (e) Spike attachment with outer capsid, (f) SARS-CoV with spikes red nucleocapsid and yellow spikes^21^; This figure was reproduced from Beniac et al.^21^, with permission; “This article is made available via the PMC Open Access Subset for unrestricted research re-use and secondary analysis in any form or by any means with acknowledgement of the original source”.

The four main operational genes encodes for spike protein (S), nucleocapsid protein (N), membrane glycoprotein (M) and a small membrane protein (SM), with an additional membrane glycoprotein (HE) occurring in the HCoV-OC43 and HKU1 beta-coronaviruses^9^. Figure 1 presents the SARS-CoV-2 structure. The virus sequence is 96% similar with a bat-related coronavirus throughout the entire genome^10^. According to WHO, no specific medicine or antiviral was found to treat or prevent novel coronavirus until now^11^. It has been noted that Chinese medication (CM), including oral administration of protective herbal formulae, the usage of CM sachets and herbal medicine fumigations, etc., is typically used to deter and manage novice coronavirus in China when the epidemic begins^11,12^. Chinese herbal and traditional medicines were also used in 2003 to combat SARS, which was the most severe infectious disease epidemic in China before COVID-19^11,13^.

## 2. Etiology

Preliminary studies show that this virus shares very high genome resemblance with 2002 bat-derived SARS coronaviruses^14^. Therefore, this virus was initially named 2019 novel coronavirus (2019-nCoV). Coronavirus is a RNA based enveloped entity named for its 9-12 nm long surface spikes for solar corona presence^15^. The coronaviral genome enclosed in the envelope bears four main structural proteins, including the spike protein (S), which binds to the Angiotensin Converting Enzyme 2 (ACE2) receptor and then mediates a fusion between the envelope and the cell membranes in the host cell to enable the virus reach to host cell^16,17^. On February 11, 2020 focused on phylogeny, taxonomy, and defining procedure, by the International Committee for Taxonomy of viruses a Coronavirus Study Group officially classified it as a severe acute respiratory syndrome coronavirus 2 (SARS-CoV-2)^18^. Shortly, WHO named the disease caused by this virus coronavirus disease 2019 (COVID-19)^19^. According to the current evidence, COVID-19 could initially be transmitted by bats and could be transferred to humans by means of pangolins^20^, or other wildlife sold on the maritime market in Huanan, but later propagated by human to human transmission^14^ , although other possibilities of transmission are not yet excluded.

## 3. Epidemiology and recent figures

The first reported patients happened in December 2019^15^. At the beginning, morbidity was very low. However, in January 2020 it hit a turning point. A significant rise in infected patients occurred in cities outside of Hubei Province during the second half of this month, due to the movement of people before the Chinese lunar New Year^22^. After an exponential rise until January 23, 2020 the infection traveled through countries and draws significant global interest. The occurrence of human-to-human infection was reported in the clusters of affected family members and medical staff^23^, by contact, droplets and fomite^10,24^. So far, there is no proof of intrauterine infection documented^25^.

**Figure 2:**
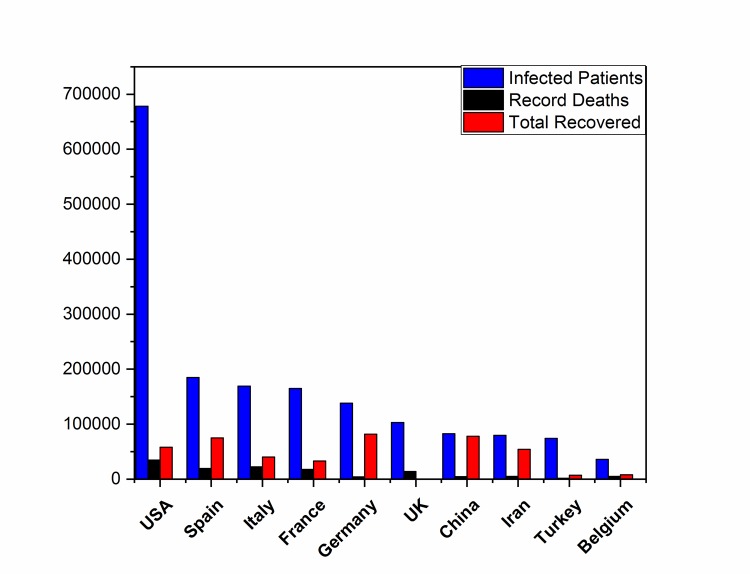
Recorded total infected people, total deaths and total recovered cases in top 10 prevalent countries Adapted from^26^.

WHO reported over 10,000 cases of COVID-19 infections across China in late January 2020^15^. On 13 February 2020, 13332 new cases were registered from Hubei for the first time. National Health Commission of China in its diagnosis and treatment program (trial fifth version) recommended chest CT diagnosis for clinical confirmation of infection in suspected cases^15^. By February 19, 2020, overall reported cases were 74,280 in China and 924 in other 24 countries, with 2009 worldwide deaths^23^. On April 17, 2020, a total of 2,200,358 infected cases were reported all over the world with 1,494,415 active cases and 705,907 closed cases. Of the total active cases, 1,437,938 (96%) were foundto have mild symptoms, and 56,477 (4%) were tagged as serious or critical cases. Among closed cases, 558,168 (79%) are recovered to their normal conditions and discharged, while 147,787 (21%) deaths occurred (Table 1 and Figure 2)^26^.

**Table 1:** Statistics by WHO; accessed on April 17, 2020

Active cases	Closed cases
1,494,415 currently infected patients	705,907 cases which had an outcome
1,437, 938 (96%) mild conditions	558,168 (79%) recovered cases
56,477 (4%) critical cases	147,787 (21%) deaths occurred

## 4. Incubation period for COVID-19 patients

The virus is believed to have incubation periods ranging between approximately 2-14 days (time from infection to symptoms) on the basis of the following sources:

1. Incubation time for COVID-19 between 2 and 10 days has been recorded by the WHO^27^.

2. The Chinese National Health Commission initially expected a 10 to 14 days incubation period^28^.

3. The Centers for Disease Control and Prevention (CDC) from United States of America assumes the incubation period of 2 to 14 days for COVID-19^29^.

4. Doctors and health-care practitioners, of leading Chinese group DXY.cn predicts an incubation period of "3 to 7 days, and up to 14 days."

In a report released on February 9, 2020, the incubation time was observed as long as 24 days (range from 0-24 days; average of 3 days)^30^. The WHO stated on February 10, 2020, at its press conference, that a very long duration of incubation can represent double exposure, 24 days was a specified point to be regarded in the sense of the study's key results^31^. Nevertheless, more recently, in a JAMA report released on February 21, 2020, a 19-day incubation period in infected patient has been identified^30^, Hubei Province registered another case with an incubation time of 27 days, on February 22, 2020^32^. The incubation time period has averaged 5.2 days. However, it greatly differs between the patients, according to a Chinese article reported by New England Journal of Medicine on January 30, 2020^28^. An analysis sponsored by the Holland Ministry of Health and released by Eurosurveillance examined data for 88 identified travelers to and from Wuhan, which were identified as COVID-19 infected patients between January 20 and 28, 2020. It was calculated that the mean incubation period was 6.4 days. The incubation duration varied from 2.1 to 11.1 days. The 11.1-day maximum limit may be known as conservative^28^. A comparison with other viruses can be found in Table 2.

**Table 2:** Comparison with other viruses Adapted from^33^

Serial no.	Virus	Incubation period
1	Novel Coronavirus COVID-19	2-14 or 0-24
2	SARS	2-7 days, as long as 10 days
3	MERS	5 days, range 2-14
4	Swine Flu	1-4 days, as long as 7 days
5	Seasonal Flu	2 days, 1-4 range

## 5. COVID-19 Mortality rate

WHO Director, general dr. Tedros Adhanom Ghebreyesus, said in his inaugural remarks at the COVID-19 press briefing on March 3, 2020, that there is a 3.4% mortality rate globally. In comparison, seasonal influenza normally kills fewer than 1% of affected individuals^34^. Initially, at a press conference on Wednesday, January 29, 2020, and again on February 10, 2020, the WHO listed 2% as a mortality rate estimation. However, on January 29, 2020, WHO specified that this was a very early and provisional estimate, that might have changed^28^. On February 20, out of 55,924 laboratory reported cases, were 2,114 reported deaths, with a case fatality rate of 3.8%^35^. National Health Commission in China released on February 4, 2020, the following figures^36^.

1. Mortality rate was found 4% in Wuhan.

2. Fatality rate in other provinces was 0.16%

3. Mortality rate was 3.1% in the Hubei province

4. 97% of the China total death by COVID-19 were in the Hubei province.

5. Mortality rate nationwide was 2.1%.

Elderly patients with specific illnesses are considered at higher risk, regardless of whether they have a coronavirus or not^36^. Patients with heart disease, high blood pressure or diabetes treated with ACE2-increasing drugs may have a high risk for serious COVID-19 infection^37^. A mortality rate comparison with other viruses is shown in Table 3, while the COVID-19 fatality rate by age, sex and with comorbidity is shown in Table 4, Table 5 and Table 6, respectively.

**Table 3:** Mortality rate comparison with other viruses Adapted from^36^.

Serial no.	Virus	Death Rate
1	SARS-CoV-2	2%
2	SARS	9.6%
3	MERS	34%
4	Swine Flu	0.02%

**Table 4:** COVID-19 fatality rate by age Adapted from^38^.

Serial.no.	Age	Death rate confirmed cases	Death rate all cases
1	80+ years old	21.9%	14.8%
2	70-79 years old	-	8.0%
3	60-69 years old	-	3.6%
4	50-59 years old	-	1.3%
5	40-49 years old	-	0.4%
6	30-39 years old	-	0.2%
7	20-29 years old	-	0.2%
8	10-19 years old	-	0.2%
9	0-9 years old	-	No fatalities

**Table 5:** COVID-19 fatality rate by sex Adopted from^38^.

Serial.no.	Sex	Death rate confirmed cases	Death rate all cases
1	Male	4.7%	2.8%
2	Female	2.8%	1.7%
			

**Table 6:** COVID-19 fatality rate with comorbidity Adopted from^38^.

Serial.no.	Pre-existing conditions	Death rate confirmed cases	Death rate all cases
1	Cardiovascular disease	13.2%	10.5%
2	Diabetes	9.2%	7.3%
3	Hypertension	8.4%	6.0%
4	Chronic respiratory disease	8.0%	6.3%
5	Cancer	7.6%	5.6%
6	No pre-existing conditions	-	0.9%

## 6. COVID-19 symptoms

Typically, COVID-19 triggers flu-like symptoms, such as fever and cough. These symptoms can develop into pneumonia, chest strain, chest pain, and difficulty to breath in elderly people and other patients who have other chronic health conditions. It looks like it begins with a fever and leads to dry coughing. One week after infection the conditions become harsh and lead to breath shortening with approximately 20% of patients requiring hospital treatment and medication^29^. The COVID-19 illness appears to seldom induce runny nose, sneezing or sore throat (only around 5% of patients have such symptoms). Painful throat sneezing, and stuffy nose are typically symptoms of seasonal flu or cold^39,40^. A few patients may develop pain or hemoptysis and many may also be completely asymptomatic^24,41^. Older people with comorbidity and serious alveolar injury are more prone to experience respiratory failure^42^. The onset of disease will demonstrate rapid progression to organ dysfunction (e.g., acute kidney injury, shock, acute cardiac injury, acute respiratory distress syndrome ARDS) and death in severe cases^41^. Sometime patients might develop lower or normal white blood cell count, thrombocytopenia or lymphopenia, with increased C-reactive protein level and extended activated thromboplastin time^24,41,42^. 80% of infected cases are found to be mild, with normal fever and flu and the patients can recover at home. In short, a patient having upper respiratory tract symptoms and fever with leukopenia or lymphopenia should be suspected (Table 7, Table 8, Table 9).

**Table 7:** COVID-19 common symptoms Adapted from^41^.

Serial.no	Symptoms	%
1	Fever	98.6%
2	Fatigue	69.6%
3	Dry Cough	59.4%

**Table 8:** COVID-19 symptoms from study of Huang et al.^1^ Adapted from^1^.

Serial no.	Common symptoms	%
1	Fever	98%
2	Cough	76%
3	Muscle pain or Fatigue	44%
Serial no.	Less common symptoms	%
1	Sputum production	28%
2	Headache	8%
3	Hemoptysis	5%
4	Diarrhea	3%

**Table 9:** COVID-19 symptoms from study of Chen et al.^42^ Reproduced from^42^ with permission.

Serial no.	Signs and Symptoms	%
1	Fever	83%
2	Cough	82%
3	Shortness of breath	31%
4	Muscle ache	11%
5	Confusion	9%
6	Headache	8%
7	Sore throat	5%
8	Runny nose	4%
9	Chest pain	2%
10	Diarrhea	2%
11	Vomiting	1%
12	More than one symptom	90%
13	Fever, cough and shortness of breath	15%

## 7. Treatment options for SARS-CoV-2 infection

To date, there are no proven effective antiviral therapies for the infection caused by SARS-CoV-2. Treatment in several hospitals includes the use of prophylactic antibiotics to prevent secondary infection. Initial reports have shown that some antivirals with antibiotics combination can be given orally with benefits^1^. On January 25, 2020 a joint research team from the Shanghai Institute of Materia Medica and Shanghai Tech University conducted a silicon drug screening and enzyme activity testing and reported 30 agents with significant antiviral activity against SARS-CoV-2^43^. The following are some drugs which have been used against COVID-19 in-vitro. Their use in the clinic is still under debate at this time.

### **7.1. Chloroquine as a general antiviral agent**

Chloroquine, a commonly used anti-malarial and autoimmune medication, has recently been identified as a possible broad spectrum antiviral drug^44,45^. In vitro, chloroquine is reported to be a variable bioactive agent that has antiviral activity against RNA viruses, such as rabies virus^46^ poliovirus^47^, HIV^48,49^ and hepatitis C virus^50^.

Chloroquine is known to prevent infection of the cells by the virus through increasing the endosomal pH needed for virus for cell fusion, and interfering with the glycosylation of SARS-CoV cell receptors^51^.

#### 7.1.1 Specific antiviral activity of Chloroquine against COVID-19

The China National Center for Biotechnology Development recently reported that chloroquine is one of the three drugs with a promising profile against the current SARS-CoV-2. Chloroquine remodeling was explored in hospitals in Beijing, in central China’s Hunan Province and South China’s Guangdong Province^52^. The drug that could potentially prevent SARS-COV-2 infection at low molecular concentrations is chloroquine, with a half-maximal effective concentration (EC50) of 1.13 μM and a half-cytotoxic concentration (CC50) greater than 100 μm^53^. However, in treating malaria, there are minor risks for adverse events, such as macular retinopathy and cardiomyopathy, whichare side effects caused by long term use of chloroquine^54-56^. Evidently, chloroquine could be used for the treatment of COVID-19 because of its effectiveness and safety for long term use^57^. A narrative letter by Chinese authors reported that chloroquine phosphate used in many clinical trials has marked efficacy against COVID-19. After oral administration, chloroquine is distributed widely across the body, including the lung. The EC90 value in Vero E6 cells was 6.90 μM, that can be clinically attainable in patients with rheumatoid arthritis who received 500 mg administration, which is demonstrated in their plasma^53^. Hydroxychloroquine, which is a derivative of chloroquine, may have lower adverse effects than chloroquine^58^.

#### 7.1.2 Ethical issues regarding the use of Chloroquine against the COVID-19

Administration of chloroquine against COVID-19 is experimental. Therefore, ethical trial approval is necessary, and ethically justifiable as the best treatment available (i.e. off-label). Additional information on chloroquine’s effects and usefulness for the treatment of COVID-19 patients will soon be published due to its experimental use in the emerging outbreak. Due to the high number of patients infected and the lack of approved drugs, timely release of this information may be critical. The WHO confirms that there is currently no evidence from randomized control trials to warn about specific COVID-19 drug treatments and that unlicensed therapies can only be performed in the sense of ethically-approved clinical trials or under strict supervision of the Controlled Emergency Use of Unregistered Procedures System (MEURI). Meanwhile, the recommendations for “Clinical management of severe acute respiratory infection when novel coronavirus (SARS-COV-2) infection is suspected”^59^. The authors tend to agree with this viewpoint of WHO to view chloroquine as experimental. The outbreak is not the perfect environment for doing so, but even the use of chloroquine off-label can be followed by many concerns; the first being the health of patients, which should be followed by close supervision. The ethical approach to off-label drug use also varies from country to country, raising concerns regarding equity. Off-label drug use could cause severe medication shortages when needed for malaria, since chloroquine remains a crucial medication in the treatment of malaria in many parts of the world^60^.

#### 7.1.3 Specific precautions before using chloroquine against COVID-19

For the use of chloroquine phosphate an expert consensus was published on February 20, 2020, by a multicenter collaboration group of the Department of Science and Technology of Guangdong Province and Health Commission of Guangdong Province. Preliminary measures recommended by the panel include blood tests to rule out the risk of anemia, thrombocytopenia or leukopenia, as well as serum electrolyte abnormalities and/or hepatic and renal function dysfunctions. It is recommended to rule out the development of QT interval prolongation or bradycardia and to perform patient interviews to find out visual and/or mental disturbance. Electrocardiography was routinely recommended and quinolones, macrolides, ondansetron as well as various antiarrhythmic, antidepressant and antipsychotic drugs which prolong the QT interval should be avoided as recommended by the panel^61^.

### 7.2. Remdesivir

Remedsivir is an analogue of adenosine, which incorporates into nascent viral RNA chains resulting in pre-mature termination. Recently, it was recognized as a potential antiviral medication against a broad variety of RNA viruses (including SARS / MERS-CoV) in cultivated cells, mice and non-human primates (NHP) models^62-64^. The EC90 value of remdesivir in Vero E6 cells against SARS-CoV-2 was 1.76 μM, indicating that its working concentration is likely to be attained in NHP models. The remdesivir has also effectively inhibited virus infection in a human cell line (Huh-7 cells of human liver cancer) that is susceptible to SARS-CoV-2^65^. Animal experiments showed that remdesivir can significantly reduce the viral load of MERS-CoV in the lung tissue of infected mice, enhance lung function and minimize pathological damage to the lung tissue^66^.

### 7.3. Favipiravir

On February 15, 2020, in China, favipiravir was approved for the treatment of the 2019 novel influenza. Currently, this drug undergoes clinical trials to treat COVID-19. Favipiravir is an inhibitor of a new type of RNA-dependent polymerase RNA (RdRp)^67^. The value of Favipiravir EC50 in Vero E6 cells (cells used in the novel coronavirus study) was as high as 67 μM. Although more in vivo research is required to test this antiviral nucleoside, it has also been shown to be 100% effective in protecting mice from the Ebola virus^68^. Preliminary results from a total of 80 patients (including the experimental group and the control group) have shown that favipiravir has a more potent antiviral effect than lopinavir / ritonavir^43^.

### 7.4. Convalescent plasma and monoclonal antibodies

Convalescent plasma and monoclonal antibodies are suggested therapies for the treatment of COVID-19 patients. Convalescent plasma or immunoglobulins are used as a last resort to improve the survival rate of patients with SARS, whose condition continued to deteriorate after pulsed methylprednisolone therapy^69^. In addition, some studies found a shorter hospital stay and lower mortality in patients treated with convalescent plasma^70^. Further probability includes Leronlimab, a humanized monoclonal antibody (CCR5 antagonist), and a nucleoside RNA polymerase inhibitor galidesivir, both of which have shown survival benefits in many deadly virus infections and are considered potential useful candidates for treatment^71,72^.

### 7.5. HIV protease inhibitors as potent antiviral against the SARS-CoV-2

Lopinavir and Ritonavir are both HIV protease inhibitors that suppress the cleavage of a polyprotein into multiple functional proteins. At the Rajavithi Hospital in Thailand, the infectious disease team used a combination of oseltamivir (anti-influenza agent) and lopinavir/ritonavir to successfully improve the status of patients with severe conditions^73^.

### 7.6. Baricitinib as suggested antiviral against SARS-CoV-2

Baricitinib, used for the treatment of rheumatoid arthritis, is an inhibitor of AAK1 and Janus kinase and is recommended to control viral replication^74^. Machine learning models predicted that AP2-associated protein kinase 1 (AAK1) drugs that disrupt these proteins can inhibit viral entry into the target cells^75^. The use of baricitinib in susceptible COVID-19-associated patients with ongoing pneumonia should be taken with strict caution^76^.

### 7.7. Drugs under clinical trials for COVID-19

Clinical trials presently focus on the efficacy of different drugs, such as immunoglobulins, arbidol hydrochloride combined with interferon atomization, ritonavir plus oseltamivir, ASC09F plus oseltamivir, mesenchymal stem cell treatment, lopinavir plus ritonavir, hydroxychloroquine, darunavir plus cobicistat, methylprednisolone and washed microbiota transplantation^65,77^. Repurposing these available drugs for immediate use in treatment of SARS-CoV-2 infections could improve the available clinical management^78^. Study of Xiaoling Xu et al. suggests that tocilizumab is an important therapy in serious COVID-19 patients, which offered a new therapeutic approach to this deadly infectious disease^79^.

## 8. Conclusion

The virus SARS-CoV-2 is a fatal disease having a high mortality rate with a total of 2,200,358 certified cases and 147,787 deaths recorded all over the world. To date, there is no efficacious, proven medicinal therapy. A committee of US experts has developed treatment guidelines for COVID-19 patients which are regularly updated^80^.

A model drug, chloroquine, proposed by WHO could potentially work against the novel coronavirus. Remdesvir, a nucleoside analogue, it also holds promise for the use in treating COVID-19 patients. There are additional proposed drugs against COVID-19, such as drugs approved by FDA for the treatment of other pathologies, including ribavirin, penciclovir, nitazoxanide, nafamostat, chloroquine and two well-known drugs having broad spectrum activity i.e. remdesivir (GS5734) and favipiravir (T-705). Leronlimab, a humanised monoclonal antibody (CCR5 antagonist) arbidol hydrochloride combined with interferon atomisation, lopinavir plus ritonavir, ritonavir plus oseltamivir, ASC09F plus oseltamivir, mesenchymal stem cell treatment could also be a choice for the treatment of COVID-19 patients. Lopinavir and Ritonavir are both HIV protease inhibitors that suppress the cleavage of a polyprotein into multiple functional proteins. Based on historical records and human reports of SARS and prevention of H1N1 influenza, Chinese herbal formula may be an effective solution to COVID-19 prevention in high-risk populations, although their usefulness in the clinic for this purpose still has to be demonstrated. In short, SARS-CoV-2 is a highly transmissible virus and clinical trials are required for finding and confirming promising drug candidates and effective vaccines.
